# Co-Administration of Amiodarone Increases Bleeding by Affecting Rivaroxaban Pharmacokinetics in Patients with Atrial Fibrillation

**DOI:** 10.3390/pharmaceutics16081006

**Published:** 2024-07-30

**Authors:** Huamin Ding, Zi Wang, Jia Wang, Yao Yao, Chi Zhang, Houwen Lin, Yong Zhou, Zhichun Gu, Qianzhou Lv, Xiaoye Li

**Affiliations:** 1 Department of Pharmacy, Punan Branch of Renji Hospital, Shanghai Jiao Tong University School of Medicine, Shanghai 200125, China; huaminding1987@163.com (H.D.); wjia2018@126.com (J.W.); zchzhangchi@163.com (C.Z.); congenital@hotmail.com (Y.Z.); 2 Department of Pharmacy, Zhongshan Hospital, Fudan University, Shanghai 200032, China; wang.zi@zs-hospital.sh.cn (Z.W.); sophieyao119@gmail.com (Y.Y.);; 3 Department of Pharmacy, Ren Ji Hospital, Shanghai Jiao Tong University School of Medicine, Shanghai 200127, China; linhouwenrenji@163.com; 4 College of Clinical Pharmacy, Shanghai Jiao Tong University School of Medicine, Shanghai 200127, China

**Keywords:** rivaroxaban, amiodarone, DDI, PK/PD, bleeding events

## Abstract

This study aimed to investigate the impact of the drug–drug interaction between rivaroxaban and amiodarone on the clinical outcomes in patients with non-valvular atrial fibrillation (NVAF), focusing on pharmacokinetic and pharmacodynamic (PK/PD) aspects. A prospective study enrolling 174 patients with NVAF who were treated with rivaroxaban was conducted. The patients were divided into two groups based on postoperative antiarrhythmic and anticoagulation strategies: the rivaroxaban group (Control group) and the rivaroxaban plus amiodarone group (Riv/Amio group). The trough plasma concentrations (C_trough_) of rivaroxaban, activated partial thromboplastin time (APTT), prothrombin time (PT), and the clinical outcomes between the two groups were compared. Patients receiving 20 mg of rivaroxaban in the Riv/Amio group had a higher concentration of rivaroxaban C_trough_ than those in the Control group (*p* = 0.009). Furthermore, in patients with moderate to severe renal impairment, rivaroxaban C_trough_ was significantly increased in the Riv/Amio group. There was no significant difference in PT and APTT between the two groups. Regarding the clinical outcomes, the combination of rivaroxaban and amiodarone medication was associated with a higher incidence of bleeding events (*p* = 0.041; HR = 2.83, 95% CI 1.05–7.66) and clinically relevant non-major bleeding (*p* = 0.021; HR = 3.65, 95% CI 1.21–10.94). Finally, independent risk factors for bleeding in NAVF patients treated with rivaroxaban were identified as its combination with amiodarone (*p* = 0.044; OR = 2.871, 95% CI 1.028–8.023). The combination of rivaroxaban and amiodarone led to changes in rivaroxaban pharmacokinetics and an elevated risk of bleeding events. Therefore, physicians prescribing rivaroxaban medications should assess the potential bleeding risk associated with the concurrent use of amiodarone, particularly in patients with renal impairment.

## 1. Introduction

Non-valvular atrial fibrillation (NVAF) is an arrhythmia that significantly increases the risk of stroke. Rivaroxaban has emerged as an effective anticoagulant for preventing thromboembolic events in patients with NVAF [1,2]. Its fixed dosing and favorable pharmacological profiles have contributed to its increasing clinical use [3,4,5]. In addition to thrombosis prevention, rhythm control is considered a crucial strategy for managing NVAF. Recent studies have suggested that rhythm control is associated with a lower risk of stroke or death [6,7,8]. According to national guidelines, amiodarone has been recommended for arrhythmia control as a second-line agent in selected patients with NVAF [9,10]. Consequently, the concurrent use of rivaroxaban and amiodarone is common in the NVAF population.

Rivaroxaban is metabolized by the hepatic enzyme cytochrome P450 3A4 (CYP3A4) and is also a substrate of permeability-glycoprotein (P-gp). Amiodarone, on the other hand, is a weak inhibitor of CYP3A4 and a potent inhibitor of P-gp. Since both drugs share absorption, metabolic, and elimination pathways, combining rivaroxaban and amiodarone is expected to increase rivaroxaban plasma concentrations and bleeding risk [11,12]. However, the findings from the ROCKET AF clinical study suggested that the co-administration of rivaroxaban and amiodarone may not be clinically relevant in patients with NVAF [13,14]. Moreover, both the European Heart Rhythm Association (EHRA) guidelines and the Food and Drug Administration do not explicitly prohibit the concomitant use of rivaroxaban and amiodarone [15,16]. Nevertheless, two retrospective cohort studies have highlighted a higher risk of bleeding in individuals prescribed medication with rivaroxaban and amiodarone compared to those taking rivaroxaban alone, conflicting with clinical data [17]. Real-world studies evaluating the pharmacokinetic (PK) and pharmacodynamic (PD) effects of rivaroxaban in combination with amiodarone have been limited.

Therefore, we conducted this prospective cohort study to comprehensively compare the efficacy and safety as affected by PKs and PDs in NVAF medicated with rivaroxaban and amiodarone. Furthermore, we performed subgroup analyses based on different ages, thrombotic scores, and rivaroxaban dosages to offer more detailed real-world evidence on the effects of combining rivaroxaban and amiodarone.

## 2. Methods

### 2.1. Patients

The patients with NVAF with indications for catheter ablation (CA) and undergoing CA at Zhongshan Hospital, Fudan University, were consecutively included. The diagnosis of NVAF adhered to the criteria outlined by the European Society of Cardiology (ESC) [18]. According to the postoperative antiarrhythmic and anticoagulation strategies, the patients were divided into a rivaroxaban group (Control group) and a rivaroxaban plus amiodarone group (Riv/Amio group). Both rivaroxaban and amiodarone are administered orally. All the participants were treated with rivaroxaban for a minimum of four consecutive weeks to ensure a stable anticoagulation state. The dosage of rivaroxaban and the need for amiodarone treatment are determined based on the specific conditions of the patients and the recommendations of their doctors. Ultimately, this prospective cohort study enrolled 175 patients (Control group: *n* = 112; Riv/Amio group: *n* = 62) between March 2022 and December 2022.

The exclusion criteria were as follows: (1) NVAF with valvular heart disease, including mechanical valve replacement or moderate to severe mitral stenosis (usually rheumatic); (2) severe renal dysfunction (creatinine clearance < 15 mL/min); (3) severe hepatic dysfunction (Child-Pugh B or C); (4) the concomitant use of other anticoagulant and antiplatelet drugs; (5) treatment with other strong inhibitors of CYP3A4 or P-gp; (6) patients with poor medication compliance or lost to follow-up.

### 2.2. Plasma Collection

After the rivaroxaban reached a steady state without dose adjustments, venous blood samples were collected in heparinized preprocessed tubes to detect the PK and PD parameters. Blood samples were collected following the last 18–26 h of dosing to determine the trough rivaroxaban concentration (C_trough_). The PD tests, including those for activated partial thromboplastin time (APTT) and prothrombin time (PT), were conducted within 2 h of sampling in the hospital laboratory department, which acquired ISO 15189 certification (Medical laboratories-requirements for quality and competence. International Organization for Standardization: Geneva, Switzerland, 2022). The plasma samples were stored at −20 °C for concentration measurements within 2 months.

### 2.3. Analytical Techniques or Procedures

The plasma concentration of rivaroxaban was determined by ultra-performance liquid chromatography with tandem mass spectrometry. The internal standard was Rivaroxaban-d4 (R538002, Toronto Research Chemicals Inc., North York, ON, Canada), and the calibration range of the assay ranged from 1 ng/mL (the lower limit of quantification) to 1000 ng/mL. The assay had 86–114% inter- and intra-accuracy and <10% inter- and intra-day precision.

The PT was measured using Thomborel^®^S (Siemens, Erlangen, Germany) on a CN600 coagulation analyzer (TOA Medical Electronic Co., Kobe, Japan). The reports ranged from 0 to 170 s.

### 2.4. Clinical Outcomes

The clinical outcomes included thromboembolic and bleeding events recorded during follow-up. Thromboembolic events encompassed stroke, pulmonary embolism, venous thromboembolism, and cardiac embolism. The definitions of major bleeding and clinically relevant non-major bleeding were based on the International Society for Thrombosis and Hemostasis (ISTH) [19]. Major bleeding was defined as meeting any of the following criteria: hemoglobin decline ≥ 2 g/dL; blood transfusion of whole blood or concentrated red blood cell ≥ 2 units; or bleeding in critical areas: intracranial, intraspinal, intraocular, pericardium, articular cavity, or retroperitoneum bleeding or intramuscular hemorrhage in compartment syndrome. Clinically relevant non-major bleeding was defined as bleeding associated with medical or physician intervention or impacting activities of daily living due to pain.

### 2.5. Statistical Analysis

Continuous variables were presented using means ± SD or medians ± interquartile ranges and tested using Student’s *t*-test or the Mann–Whitney U-test, depending on the normality of distribution assessed using the Shapiro–Wilk test in IBM SPSS 23 software. Categorical variables were expressed as percentages (%) and compared using the χ^2^ or Fisher’s exact test. Kaplan–Meier curves were utilized to display the cumulative clinical outcomes, and Log-rank tests were employed to calculate hazard ratios, confidence intervals, and *p*-values. Baseline variables considered clinically relevant and candidate variables with a *p*-value < 0.2 on univariate analysis were included in the multivariable model. Given the number of events available, variables for inclusion were carefully chosen to ensure the parsimony of the final model. All the statistical analyses were performed using SPSS 23 for Macintosh (IBM, Armonk, NY, USA), and the data analysis and plotting were carried out using R. Statistical significance was defined as *p* < 0.05.

## 3. Results

### 3.1. Patient Characteristics

The baseline demographic characteristics of the participants are included in Table 1. In the Control group, 40.2% were female, with an average age of 67. The mean CHA2DS2-VASc score and HAS-BLED score were 2.63 and 2.29, respectively. The Riv/Amio group had an average age of 66 years, with 32.3% of them being female. The mean CHA2DS2-VASc score was 2.92, and the HAS-BLED score was 2.40. The baseline comorbidities were similar between the two groups. Baseline comorbidities, history of stroke or bleeding, renal function, and different dosages of rivaroxaban were similar between the two groups. Notably, the history of catheter radiofrequency ablation differed significantly between the groups, with 4.5% in the Control group and 21.5% in the Riv/Amio group.

### 3.2. Effects of PD and PK Parameters of Concurrent Medication of Rivaroxaban with Amiodarone

**Figure 1:**
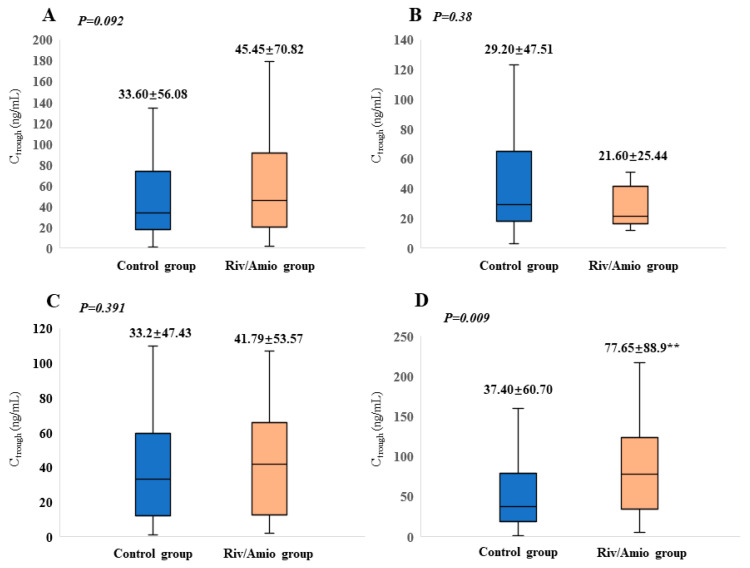
Impact of amiodarone co-administration on the trough levels of rivaroxaban. (**A**) Entire dosage rivaroxaban group; (**B**) rivaroxaban 10 mg; (**C**) rivaroxaban 15 mg; (**D**) rivaroxaban 20 mg; Control group: rivaroxaban group; Riv/Amio group: rivaroxaban plus amiodarone group. ** represents *p <* 0.01.

**Figure 2:**
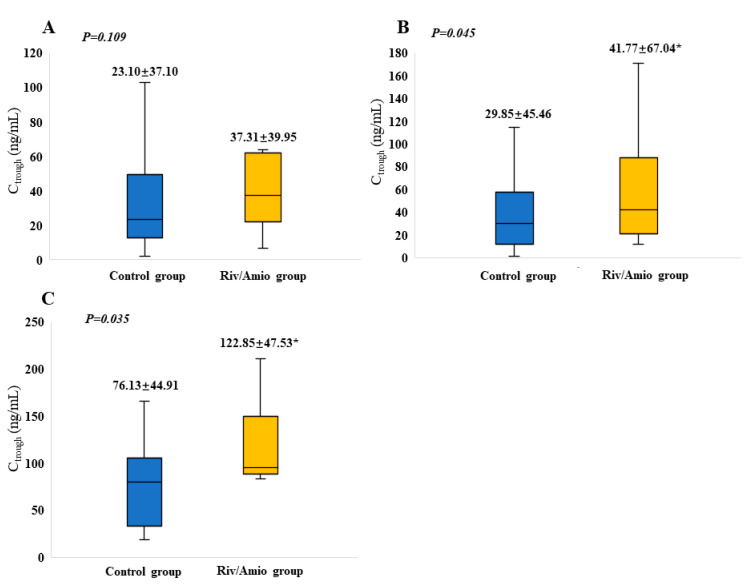
Impact of amiodarone co-administration on the trough levels of rivaroxaban in patients with renal impairment. (**A**) Normal renal function (estimated glomerular filtration rate (eGFR) 90~120 mL/min/1.73 m^2^); (**B**) mild renal impairment (eGFR 60~89 mL/min/1.73 m^2^); (**C**) moderate to severe renal impairment (eGFR 15~59 mL/min/1.73 m^2^); Control group: rivaroxaban group; Riv/Amio group: rivaroxaban plus amiodarone group. * represents *p <* 0.05.

The PD profiles are summarized in Table 2. There was no significant difference in PT (*p* = 0.685) or APTT (*p* = 0.662) between the patients using rivaroxaban and amiodarone concurrently and those using rivaroxaban alone. This consistency persisted when analyzing patients aged ≥65, with a CHA2DS2-VASc score ≥ 3, or with moderate to severe renal impairment. The findings regarding the effect of drug–drug interaction (DDI) on the rivaroxaban PKs are shown in Figure 1. No significant differences in C_trough_ were found between the rivaroxaban group and the combination drug group (*p* = 0.092). However, amiodarone notably increased the C_trough_ of rivaroxaban in the patients treated with 20 mg of rivaroxaban (*p* = 0.009). The impact of renal impairment on the C_trough_ of rivaroxaban is depicted in Figure 2. Significantly increased PK parameters were observed in patients with mild renal impairment (*p* = 0.045) and moderate to severe renal impairment (*p* = 0.035).

### 3.3. Influence of Combined Administration of Amiodarone and Rivaroxaban on Clinical Outcomes

**Figure 3:**
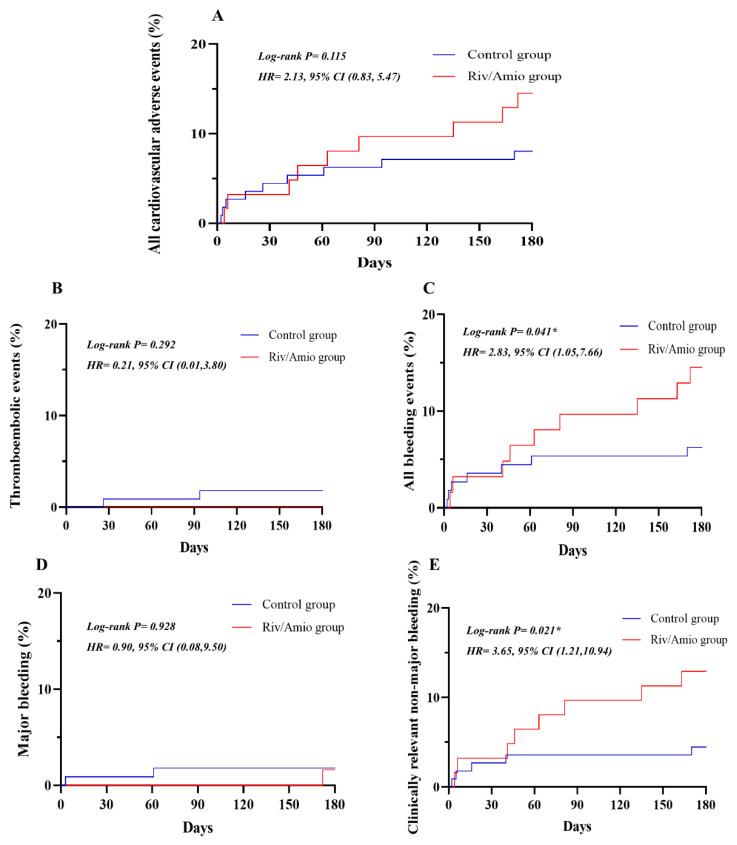
Influence of combined administration of amiodarone and rivaroxaban on clinical outcomes, included all cardiovascular adverse events (**A**), thromboembolic events (**B**), all bleeding events (**C**), major bleeding (**D**), clinically relevant non-major bleeding (**E**). All cardiovascular adverse events contained thromboembolic and any bleeding events. Control group: rivaroxaban group; Riv/Amio group: rivaroxaban plus amiodarone group. * represents *p <* 0.05.

During the 180-day follow-up period, nine patients in the Control group experienced cardiovascular adverse events, comprising two thromboembolic events and seven bleeding events. All 10 patients in the Riv/Amio group experienced bleeding events (Table 3). Although there was no significant difference in the thromboembolic events between the two groups (1.79% versus 0%, *p* = 0.292), no occurrences were noted in the Riv/Amio group. The Riv/Amio group exhibited significantly higher rates of bleeding events (6.25% versus 16.13%, *p* = 0.041) and clinically relevant non-major bleeding (4.46% versus 14.52%, *p* = 0.021) than the Control group. However, the two groups showed no significant difference in major bleeding events (Figure 3).

### 3.4. Combined Medication with Amiodarone Predicting the Risk of Bleeding with Rivaroxaban

Furthermore, we conducted additional analyses to investigate the associations between potential risk factors and the incidence of bleeding events (Table 4). Gender, age, body mass index, CHA2DS2-VASc score, HAS-BLED score, smoking status, amiodarone use, dyslipidemia, hypertension, diabetes, chronic kidney disease, history of bleeding, and history of stroke were initially included in the univariate logistic analysis. Subsequently, factors showing low *p*-values < 0.2 were incorporated into the multivariate logistic analysis. Amiodarone use (*p* = 0.044; OR = 2.871, 95% CI 1.028–8.023) emerged as an independent risk factor for bleeding.

## 4. Discussion

The current study offers a comprehensive exploration of the rivaroxaban–amiodarone interaction in terms of the PDs, PKs, and clinical outcomes among NVAF patients, which presents the following main findings. Firstly, the interaction did not impact PT or APTT. Secondly, the concurrent use of rivaroxaban and amiodarone correlated with increased rivaroxaban PK parameters, particularly in patients with renal impairment. Thirdly, their interaction was associated with a significantly elevated risk of bleeding, particularly clinically relevant non-major bleeding. Finally, amiodarone use and a HAS-BLED score ≥ 2 were identified as independent bleeding risk factors in rivaroxaban-treated patients.

Rivaroxaban has two pathways of elimination, namely CYP3A4 and P-gp [20]. Amiodarone is a strong P-gp inhibitor and a weak CYP3A4 inhibitor [21,22]. There is no contraindication or recommendation for amiodarone on the rivaroxaban label given that the present evidence is insufficient. The analysis based on 8% of patients in ROCKET AF who received concomitant amiodarone suggested no clinically relevant increase in rivaroxaban exposure [13]. A meta-analysis of four randomized controlled trials found no significant difference in stroke or systemic embolism, major bleeding, or intracranial bleeding in patients receiving a NOAC with or without amiodarone [14]. However, the clinical outcomes observed in real-world studies have differed from those in clinical trials. A recent study conducted on a large group of 91,330 patients who were prescribed apixaban, dabigatran, or rivaroxaban revealed that the risk of major bleeding was significantly higher in patients who also took amiodarone alongside a NOAC when compared to patients who only took a NOAC [23]. Therefore, while the potential for clinically significant interactions between amiodarone and rivaroxaban remains, further investigations are warranted to define this effect precisely.

Regarding the PK parameters, no studies have examined rivaroxaban exposure as it is affected by rivaroxaban–amiodarone DDI. Physiologically based pharmacokinetic (PBPK) modeling is a well-established approach that is used to estimate the effects of DDI on pharmacokinetics. Two PBPK models showed a small to moderate increase in rivaroxaban exposure in patients with amiodarone, which was within the pre-defined dose exposure equivalence range, so it was unlikely to have any significant clinical implications [24,25]. Importantly, given that renal insufficiency produced clinically significant increases in rivaroxaban exposure with amiodarone co-administration, the FDA label warns that renally impaired patients taking rivaroxaban with P-glycoprotein inhibitors and weak to moderate CYP3A4 inhibitors (such as amiodarone) may have a higher risk of bleeding. The PBPK modeling indicated a larger increase in the area under the curve for rivaroxaban plasma concentration–time in patients with mild or moderate renal impairment [24]. Similarly, in our study, the C_trough_ of rivaroxaban was significantly increased in patients with moderate to severe renal impairment. Therefore, a greater benefit versus risk can be achieved with rivaroxaban dose reductions in renally impaired subjects, with an estimated glomerular filtration rate of 15–59 mL/min/1.73 m^2^ with concomitant amiodarone and rivaroxaban use. The effect of rivaroxaban on the PT and APTT has been assessed in multiple studies [26,27,28,29]. PT and APTT are prolonged by rivaroxaban, and rapid preliminary screening of excessive anticoagulation and bleeding risk has been recommended for application in the absence of blood concentration monitoring in this context. However, the concentration-dependent correlation was generally weak and became weaker with increasing concentrations of rivaroxaban [30,31,32,33,34]. The APTT assay sensitivity was also reagent-dependent. In the present study, PT and APTT did not show any difference among patients with concomitant use of rivaroxaban and amiodarone, either in the total or subgroup subjects. But more bleeding events, especially clinically relevant non-major bleeding, were observed in the Riv/Amio group. This may be due to the PT and APTT being insufficiently sensitive for assessing bleeding risk with rivaroxaban. Notably, several studies have assessed the relationship between anti-factor Xa activity and rivaroxaban [35,36]. Rivaroxaban-calibrated chromogenic anti-Xa activity assays showed linear, concentration-dependent correlations over a wide range of rivaroxaban concentrations. Therefore, anti-factor Xa activity may be a more sensitive PD parameter for bleeding risk assessment with rivaroxaban.

Our multivariate logistic analysis identified co-medication with amiodarone as an independent risk factor for bleeding. Targeted tools for assessing bleeding risk for individuals taking NOACs are expected to develop [37]. Concomitant use with amiodarone or other P-glycoprotein inhibitors or weak to moderate CYP3A4 inhibitors may be important predictors to added to tools built in the future.

This study has several limitations. Firstly, this is a single-center study with few bleeding events, which may affect the broad applicability of the results. A larger multicenter trial should be conducted to accurately assess the bleeding risks when administering rivaroxaban and amiodarone together. Secondly, the small sample size also hinders the ability to identify statistically significant differences in major bleeding events between the groups. Thirdly, the gap in the sample size between the groups could lead to inaccuracies in evaluating differences through hypothesis testing. Despite using a less sensitive statistical method, the results should be interpreted cautiously. Finally, although our study provided valuable information by measuring the trough concentrations, it did not allow for the generation of complete pharmacokinetic parameters. Future studies should be designed to include more comprehensive pharmacokinetic parameters, including peak concentration and area under the curve.

In conclusion, combining amiodarone with rivaroxaban increased the C_trough_ of rivaroxaban, further influencing the occurrence of bleeding in this prospective cohort study. The DDI of rivaroxaban and amiodarone should be carefully considered in the setting of impaired renal function. Larger studies assessing the clinical impact of DOAC drug interactions, especially in the population with a high bleeding risk, would be beneficial for patient management.

## Figures and Tables

**Table 1:** Baseline characteristics of enrolled participants.

Characteristic	Control Group (*n* = 112)	Riv/Amio Group (*n* = 62)	*p*-Value
Female sex: *n* (%)	45 (40.2%)	21 (32.3%)	0.264
Age: year	66.88 ± 10.87	66.21 ± 12.33	0.363
Weight: kg	69.90 ± 13.68	68.08 ± 14.52	0.549
Height: cm	167.11.58 ± 8.45	165.36 ± 8.86	0.615
Smoking: n (%)	19 (17.0%)	6 (9.2%)	0.260
Drinking: n (%)	12 (10.7%)	4 (6.2%)	0.422
Comorbidities: n (%)			
Hypertension	59 (52.7%)	21 (32.3%)	0.528
Diabetes	23 (20.5%)	16 (24.6%)	0.451
Dyslipidemia	8 (7.1%)	6 (9.2%)	0.571
CKD	6 (5.3%)	6 (9.2%)	0.351
CA	5 (4.5%)	14 (21.5%)	0.01
CHD	11 (9.8%)	4 (6.2%)	0.578
eGFR: n (%)			
90~120 mL/min/1.73 m^2^	31 (27.7%)	21 (33.9%)	0.217
60~89 mL/min/1.73 m^2^	52 (46.4%)	30 (48.4%)	0.413
15~59 mL/min/1.73 m^2^	24 (21.4%)	7 (11.3%)	0.076
Dosage: n (%)			
10 mg	22 (19.6%)	11 (17.7%)	0.452
15 mg	38 (33.9%)	27 (43.6%)	0.147
20 mg	51 (45.5%)	24 (38.7%)	0.224
History of bleeding: n (%)	7 (6.3%)	7 (11.3%)	0.257
History of stroke: n (%)	32 (28.6%)	21 (33.9%)	0.288
CHA_2_DS_2_-VASc score	2.63 ± 1.71	2.92 ± 1.77	0.953
HAS-BLED score	2.29 ± 1.26	2.40 ± 1.26	0.957

CKD: chronic kidney disease; CA: catheter radiofrequency ablation; CHD: coronary atherosclerotic heart disease; eGFR: estimated glomerular filtration rate; Control group: rivaroxaban group; Riv/Amio group: rivaroxaban plus amiodarone group.

**Table 2:** Impact of amiodarone co-administration on the PD parameters of rivaroxaban.

PD Parameters	Control Group	Riv/Amio Group	*p*-Value
PT: s	12.50 ± 2.00	12.50 ± 2.13	0.685
Age ≥ 65 years	12.80 ± 2.17	12.65 ± 2.21	0.761
CHA2DS2-VASc score ≥ 3	12.90 ± 2.33	12.75 ± 2.58	0.887
eGFR (15~60 mL/min/1.73 m^2^)	13.15 ± 3.5	14.4 ± 8	0.372
APTT: s	28.4 ± 6.03	28.85 ± 4.02	0.662
Age ≥ 65 years	28.96 ± 6.33	29 ± 3.75	0.529
CHA2DS2-VASc score ≥ 3	29.38 ± 8.63	29 ± 4.4	0.819
eGFR (15~60 mL/min/1.73 m^2^)	29.85 ± 4.86	30.98 ± 5.89	0.434

PD: pharmacodynamic; PT: prothrombin time; APTT: activated partial thromboplastin time; Control group: rivaroxaban group; Riv/Amio group: rivaroxaban plus amiodarone group.

**Table 3:** Influence of combined administration of amiodarone and rivaroxaban on clinical outcomes.

*n*. (%)	Control Group (*n* = 112)	Riv/Amio Group (*n* = 62)	Hazard Ratio (95% CI)	*p*-Value
All cardiovascular adverse events	9 (8.04)	10 (16.13)	2.13 (0.83, 5.47)	0.115
Thromboembolic events	2 (1.79)	0 (0.00)	0.21 (0.01, 3.80)	0.292
All bleeding events	7 (6.25)	10 (16.13)	2.83 (1.05, 7.66)	0.041 *
Major bleeding	2 (1.79)	1 (1.61)	0.90 (0.08, 9.50)	0.928
Clinically relevant non-major bleeding	5 (4.46)	9 (14.52)	3.65 (1.21, 10.94)	0.021 *

Control group: rivaroxaban group; Riv/Amio group: rivaroxaban plus amiodarone group. * represents *p <* 0.05.

**Table 4:** Univariate and multivariate analyses for the association of candidate risk factors with bleeding events.

		Univariate Analysis		Multivariate Analysis	
Variables		OR (95% CI)	*p* Value	OR (95% CI)	*p* Value
Gender	Male vs. female	0.42 (0.243, 1.805)	0.662		
Age	≥65 vs. <65	2.121 (0.661, 6.803)	0.206		
BMI	≥24 vs. <24	1.324 (0.427, 4.110)	0.627		
CHA2DS2-VASc	≥3 vs. <3	1.81 (0.638, 5.135)	0.256		
HAS-BLED score	≥2 vs. <2	1.916 (0.685, 5.348)	0.215		
Smoking	Yes vs. no	0.777 (0.166, 3.625)	0.748		
Amiodarone use	Yes vs. no	2.969 (1.069, 8.251)	0.037 *	2.871 (1.028, 8.023)	0.044 *
Dyslipidemia	Yes vs. no	1.611 (0.329, 7.889)	0.556		
Hypertension	Yes vs. no	0.715 (0.262, 1.950)	0.512		
Diabetes	Yes vs. no	2.033 (0.700, 5.906)	0.192	1.918 (0.649, 5.673)	0.239
CKD	Yes vs. no	0.830 (0.100, 6.850)	0.862		
History of bleeding	Yes vs. no	1.6 (0.327, 7.835)	0.562		
History of stroke	Yes vs. no	1.277 (0.446, 3.654)	0.649		

BMI: body mass index; CKD: chronic kidney disease. * represents *p* < 0.05.

## Data Availability

The data that support the findings of this study are available from the corresponding author upon reasonable request. The data are not publicly available due to containing clinical and personal information.
